# DLEU1 promotes cell survival by preventing DYNLL1 degradation in esophageal squamous cell carcinoma

**DOI:** 10.1186/s12967-022-03449-w

**Published:** 2022-05-26

**Authors:** Qihang Li, Zhiyu Zhang, HongChao Jiang, Jun Hou, Yuhang Chai, Hongxing Nan, Feng Li, Lianghai Wang

**Affiliations:** 1 Key Laboratory of Xinjiang Endemic and Ethnic Diseases, Shihezi University School of Medicine, Shihezi, Xinjiang China; 2 Department of Pathology and Medical Research Center, Beijing Chaoyang Hospital, Capital Medical University, Beijing, China; 3 NHC Key Laboratory of Prevention and Treatment of Central Asia High Incidence Diseases, The First Affiliated Hospital, Shihezi University School of Medicine, Shihezi, Xinjiang China

**Keywords:** lncRNA, Apoptosis, Ubiquitination, RNF114, BCL2

## Abstract

**Background:**

Emerging evidence has highlighted the critical roles of long noncoding RNAs (lncRNAs) in tumor development and progression. However, the biological functions and underlying mechanisms of DLEU1 in esophageal squamous cell carcinoma (ESCC) remain unclear.

**Methods:**

LncRNA expression in ESCC tissues was explored using lncRNA microarray datasets. The functional roles of DLEU1 in ESCC were demonstrated by a series of in vitro and in vivo experiments. RNA pull-down and immunoprecipitation assays were performed to demonstrate the potential mechanisms of DLEU1.

**Results:**

In a screen for differentially expressed lncRNAs in ESCC, we determined that DLEU1 was one of the most overexpressed lncRNAs in ESCC tissues and that upregulated DLEU1 expression was associated with a worse prognosis. Functional assays showed that DLEU1 promoted tumor growth by inhibiting cell apoptosis. Mechanistically, DLEU1 could bind and stabilize DYNLL1 by interfering with RNF114-mediated ubiquitination and proteasomal degradation. The DLEU1/DYNLL1 axis subsequently upregulated antiapoptotic BCL2 and promoted cell survival. Furthermore, DLEU1 upregulation was at least partly facilitated by promoter hypomethylation. Notably, targeting DLEU1 sensitized ESCC cells to cisplatin-induced death.

**Conclusions:**

Our findings suggest that DLEU1-mediated stabilization of DYNLL1 is critical for cell survival and that the DLEU1/DYNLL1 axis may be a promising therapeutic target for ESCC.

**Supplementary Information:**

The online version contains supplementary material available at 10.1186/s12967-022-03449-w.

## Background

Esophageal cancer is one of the most common malignancies worldwide, and it has a poor prognosis and a high mortality rate [1]. Esophageal squamous cell carcinoma (ESCC) is one of the major histological subtypes, accounting for 90% of esophageal cancer cases globally [2, 3]. The occurrence of ESCC is a complex process that includes environmental factors (such as smoking, drinking, diet, and lack of trace elements) and genetic factors (such as chromosome changes, methylation, gene polymorphism, epigenetic changes) [3–5]. Despite improvements in ESCC treatment in recent decades, the prognosis of ESCC is still unsatisfactory [6, 7]. Therefore, it is of great importance to reveal the molecular mechanism of esophageal carcinogenesis and improve therapies for ESCC patients.

Recent findings have revealed that approximately 98% of human genome transcripts are noncoding RNAs, which can be divided into long noncoding RNAs (lncRNAs) and small noncoding RNAs [8, 9]. LncRNAs, which are longer than 200 nucleotides with little or no coding potential, are involved in almost every step of regulating gene expression, such as chromatin modification and transcriptional and posttranscriptional processing [10, 11]. Aberrantly expressed lncRNAs play critical roles in the development and progression of human malignancies and can serve as tumor biomarkers and specific therapeutic targets [12–15]. However, their significance and detailed underlying mechanisms remain poorly understood in ESCC.

The lncRNA deleted in lymphocytic leukemia 1 (DLEU1) is located at 13q14.3, a region recurrently deleted in chronic lymphocytic leukemia, and it is supposed to be a tumor suppressor gene in hematopoietic tumors [16, 17]. In contrast, DLEU1 expression is elevated in colorectal cancer and head and neck squamous cell carcinoma, contributing to tumor progression [18, 19]. Nevertheless, the functional significance of DLEU1 in other tumors, especially ESCC, is unclear.

In the present study, we attempted to identify lncRNAs critical for ESCC tumorigenesis by exploring aberrant expression in lncRNA profiling datasets for paired tumor and adjacent normal tissues from two independent cohorts. We found that the expression of DLEU1 was significantly upregulated in tumor tissues and correlated with poor prognosis in ESCC patients. Functional analyses revealed that DLEU1 was required for the growth and apoptosis resistance of ESCC cells. Mechanistic studies showed that DLEU1 interfered with the degradation of DYNLL1 mediated by the E3 ubiquitin ligase RNF114, thereby enhancing the survival and tumorigenicity of ESCC cells. Our data revealed the novel antiapoptotic role of DLEU1 by mediating RNF114/DYNLL1 signaling, suggesting that DLEU1 could be a potential therapeutic target for ESCC.

## Materials and methods

### Cell lines and clinical samples

Authenticated ESCC lines TE-1 and KYSE-150 were purchased from the Cell Bank, Type Culture Collection of Chinese Academy of Sciences; KYSE-30 and KYSE-410 were obtained from the Procell Life Science & Technology; EC109 was purchased from the Cell Culture Center of the Institute of Basic Medical Sciences, Chinese Academy of Medical Sciences. All cells were cultured in RPMI-1640 medium (Gibco) supplemented with 10% fetal bovine serum (BI) at 37 °C with 5% CO_2_ in a humidified incubator. All experiments were performed with mycoplasma-free cells.

Human ESCC and paired adjacent noncancerous tissues from 75 ESCC patients who underwent esophagectomy without prior chemotherapy or radiotherapy were obtained from the First Affiliated Hospital, Shihezi University School of Medicine. Subjects were recruited with written informed consent and with approval from the Ethics Committee of the First Affiliated Hospital, Shihezi University School of Medicine.

### Quantitative real-time PCR

Total RNA was isolated from cell cultures with a total RNA kit I (Omega Bio-Tek) according to the standard protocol and reverse transcribed into cDNA using SuperQuick RT MasterMix (CWBIO). Target genes were amplified by quantitative real-time PCR (qRT-PCR) with Fast SYBR Green qPCR Master Mix (CWBIO) and specific primers using the 7500 Fast Real-Time PCR System (Applied Biosystems). GAPDH was used as an internal control. The primer sequences used for qRT-PCR are listed in Additional file 1: Table S1.

### Cell proliferation and colony formation assays

Cells seeded on 96-well plates (5000/well) were transfected with siRNAs or scramble control RNA oligos. Cell viability was measured using a Cell Counting Kit-8 (CCK-8; Dojindo) following the manufacturer’s protocol. The number of viable cells was quantified according to the absorbance at 450 nm (OD_450_) detected by a microplate reader (BIO‐RAD xMark).

Cells transfected with siRNAs or scramble control RNA oligos were plated on 6-well plates and maintained in medium containing 10% FBS for 10 days for the colony-forming assay. Colonies were fixed with methanol, stained with crystal violet, and counted.

### Cell cycle and apoptosis analyses

ESCC cells were harvested at 48 h posttransfection by centrifugation with cold PBS and then fixed in ice-cold 70% ethanol at 4 °C overnight. The fixed cells were washed twice with PBS and subjected to PI staining using the Cell Cycle and Apoptosis Analysis Kit (Beyotime Biotechnology). For cell apoptosis analysis, ESCC cells harvested after transfection were stained with the FITC Annexin V Apoptosis Detection Kit I (BD Biosciences) according to the manufacturer's instructions. Acquired data were analyzed using FlowJo software.

### RNA pull-down and mass spectrometry analysis

RNA pull-down was performed using the Pierce Magnetic RNA–Protein Pull-Down Kit (Thermo Fisher) according to the manufacturer's instructions. Biotin-labeled RNAs were transcribed in vitro with biotin RNA-labeling mix and T7 RNA polymerase (Invitrogen) following the manufacturer’s protocols. The biotinylated sense or antisense DLEU1 RNA generated in vitro was incubated with total cell lysates. Interacting proteins were isolated with streptavidin agarose beads (Invitrogen) at room temperature for 2 h. The complexes were washed briefly with washing buffer three times. The proteins precipitated with streptavidin beads were diluted in protein lysis buffer and resolved by SDS/PAGE, liquid chromatography with tandem mass spectrometry, or immunoblotting.

### Immunoblotting

Equal amounts of total cell lysates were separated by SDS-PAGE and electrophoretically transferred to PVDF membranes. The membrane was blocked with 5% skimmed milk and incubated with primary antibodies against DYNLL1 (ab51603, 1:1000; Abcam), BIM (#12933, 1:1000; Cell Signaling Technology), BCL2 (AB112, 1:1000; Beyotime Biotechnology), β‐actin (1E9A3, 1:1000; ZSGB‐BIO), and FLAG (#14793, 1:1000, Cell Signaling Technology).

### RNA in situ hybridization

Detection of DLEU1 transcripts in formalin-fixed, paraffin-embedded samples was conducted using RNAscope Probe-Hs-DLEU1 and RNAscope 2.5 High Definition Reagent Kit-RED (322350; Advanced Cell Diagnostics) according to the manufacturer's instructions. The staining process was completed by ZSGB-BIO (Beijing, China). Positive staining was indicated by red punctuate dots in the cytoplasm or nucleus. DLEU1 expression levels were categorized into five grades according to the manufacturer’s scoring guidelines: score 0, no staining or < 1 dot per 10 cells; score 1, 1–3 dots per cell (visible at 20–40× magnification); score 2, 4–9 dots per cell; score 3, 10–15 dots per cell and < 10% positive cells have dot clusters; score 4, > 15 dots per cell and > 10% positive cells have dot clusters. Cases that scored higher than 0 were considered positive.

### Immunohistochemistry

Immunohistochemical staining of DYNLL1 and BCL2 was performed using primary antibodies against DYNLL1 (ab51603, 1:600; Abcam) and BCL2 (AB112, 1:200; Beyotime Biotechnology) on tissue microarray sections by a BOND-MAX Automated IHC/ISH Stainer (Leica). The immunostaining degree of the DYNLL1 and BCL2 proteins was evaluated as previously described [20] by pathologists based on the nuclear staining intensity (intensity score) and percentage of positive cells (extent score). The final immunoreactivity score for each sample was the product of the intensity score and extent score.

### Xenograft tumor-formation assay

Four-week-old athymic female BALB/c mice were housed under specific pathogen-free conditions. For tumorigenicity determination, 1 × 10^6^ cells suspended in a volume of 0.1 ml were subcutaneously injected into the armpits of mice. The mice were sacrificed at 21 days postinjection. For treatment, mice bearing established xenografts of shDLEU1 and shCtrl cells were randomly divided into groups and intraperitoneally injected every other day for two weeks with PBS (vehicle) as a control or cisplatin (1 mg/kg body weight). Tumor volume was measured every other day and calculated using the formula length × width^2^ × π/6. All animal experimental procedures were approved by the Institutional Animal Care and Use Committee of the First Affiliated Hospital of Shihezi University School of Medicine.

### Statistical analysis

Numerical data are presented as the mean ± SEM. Statistical analysis was carried out using GraphPad Prism. Comparisons of changes between two groups were conducted using nonparametric paired or unpaired Wilcoxon tests or parametric unpaired Student's *t* tests. Comparisons between multiple groups were performed using one-way ANOVA with Tukey’s post-hoc test. Kaplan–Meier analysis and log-rank tests were used for survival estimates. *P* < 0.05 was considered to indicate statistical significance.

## Results

### DLEU1 is upregulated in ESCC and predicts poor prognosis

**Fig. 1:**
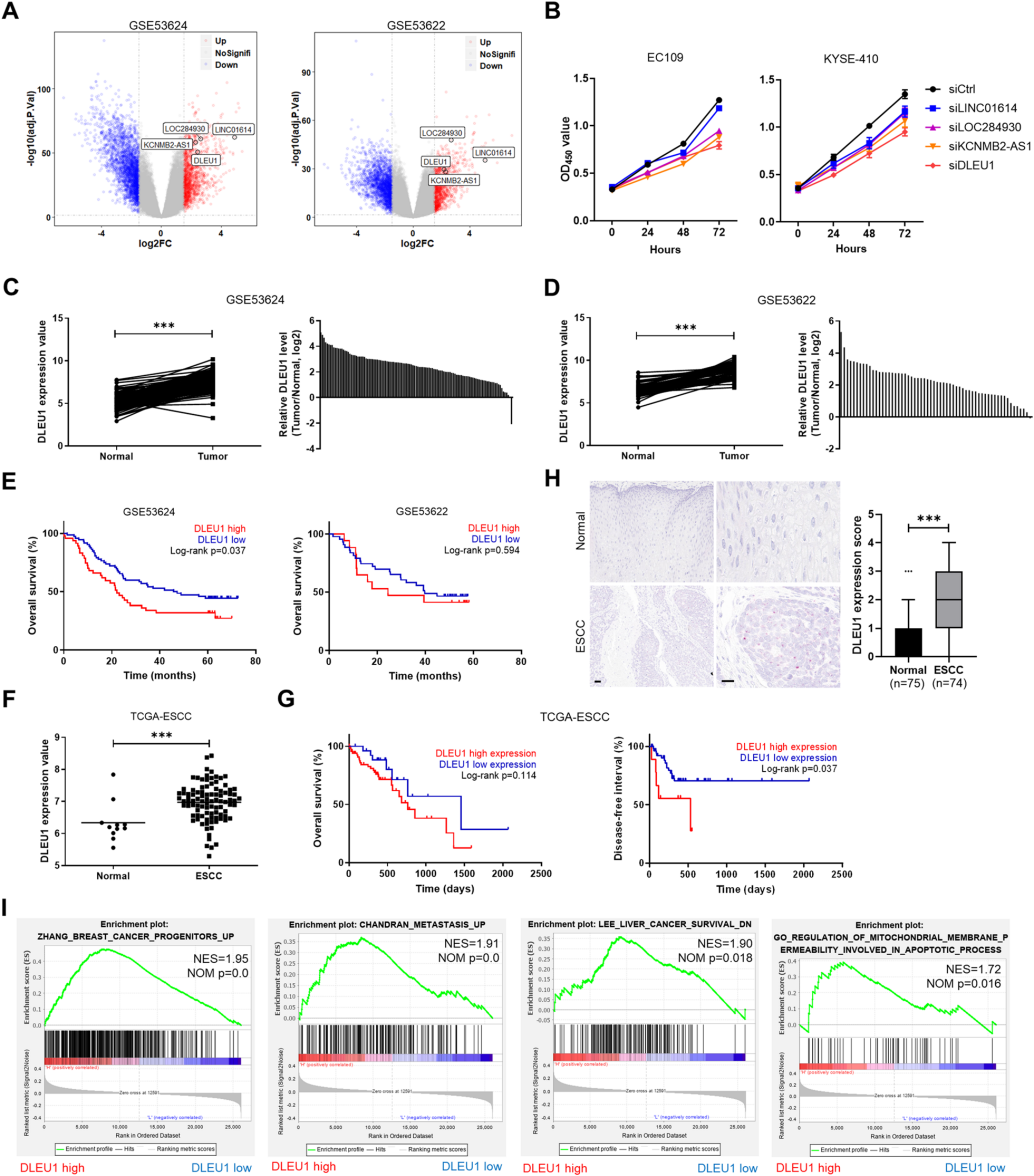
Upregulated DLEU1 expression is associated with a poor prognosis in ESCC patients. **A** Volcano plots for differentially expressed lncRNAs in ESCC based on two publicly available microarray datasets: GSE53624 (left; n = 119 pairs) and GSE53622 (right; n = 60 pairs). **B** CCK-8 analysis of the relative growth rates of EC109 (left) and KYSE-410 (right) cells transfected with the indicated siRNA. **C**, **D** Comparison of DLEU1 expression between 119 (**C**) or 60 (**D**) ESCC and paired normal tissues. **E** Kaplan–Meier curves depicting the overall survival of ESCC patients with high or low DLEU1 expression. **F** Comparison of DLEU1 expression between tumor and adjacent normal tissues from the TCGA-ESCC dataset, which was obtained from the UCSC Xena platform. **G** Kaplan–Meier survival analysis according to DLEU1 expression levels in ESCC samples from the TCGA-ESCC dataset. **H** Representative images (left) and quantification (right) of DLEU1 expression in ESCC and matched nontumor tissues by ISH staining. Scale bars, 100 μm (left) and 50 μm (right). **I** GSEA of the indicated gene sets comparing samples with high and low DLEU1 expression in the GSE53624 dataset. NES, normalized enrichment score. Significance was assessed by the Wilcoxon matched-pairs signed rank test (**C**, **D**), log-rank test (**E**, **G**), or Mann–Whitney test (**F**, **H**). ***P < 0.001

To identify lncRNAs involved in the development of ESCC, we first analyzed two independent lncRNA + mRNA microarray profiles comprising 119 and 60 pairs of ESCC samples with matched adjacent normal tissues, and screened for differentially expressed lncRNAs in ESCC (Fig. 1A). Four of the most frequently overexpressed lncRNAs in cancer tissues, namely, LINC01614, LOC284930, KCNMB2-AS1, and DLEU1, were selected for functional analysis. To evaluate their effect on cell growth, ESCC cells were transfected with a pool of three siRNAs targeting each of these four lncRNAs. Among those, we found that knocking down DLEU1 had a potent suppressive effect on ESCC cell growth (Fig. 1B). Thus, we focused on DLEU1 for further studies. By comparing the two independent cohorts, we found that DLEU1 expression was higher in 118 (99.2%) and 59 (98.3%) tumors than in their matched normal counterparts (Fig. 1C, D). ESCC patients with high DLEU1 expression tended to have decreased overall survival compared with those with low DLEU1 expression (Fig. 1E). We also observed significantly higher DLEU1 expression in tumors than in normal tissues and a correlation between DLEU1 upregulation and worse prognosis in ESCC patients from The Cancer Genome Atlas (TCGA) project (Fig. 1F, G). Moreover, overexpression of DLEU1 was positively correlated with lymph node metastasis and advanced clinical stages (Additional file 1: Table S2). RNAscope assays also confirmed upregulated DLEU1 expression in ESCC compared to adjacent nontumor tissues (Fig. 1H). To gain insight into the underlying mechanism of DLEU1 in ESCC development and progression, we conducted gene set enrichment analysis (GSEA) to identify the potential DLEU1-regulated pathways. Intriguingly, GSEA enrichment plots showed that genes upregulated in breast cancer stem cells, genes upregulated in metastatic prostate cancer, genes highly expressed in liver cancer with poor survival, and genes regulating mitochondrial membrane permeability that are involved in the apoptotic process were significantly enriched in ESCC with high DLEU1 expression levels (Fig. 1I), suggesting the potential contribution of DLEU1 to poor prognosis and mitochondrial apoptosis.

### DLEU1 promotes tumor growth by inhibiting cell apoptosis

**Fig. 2:**
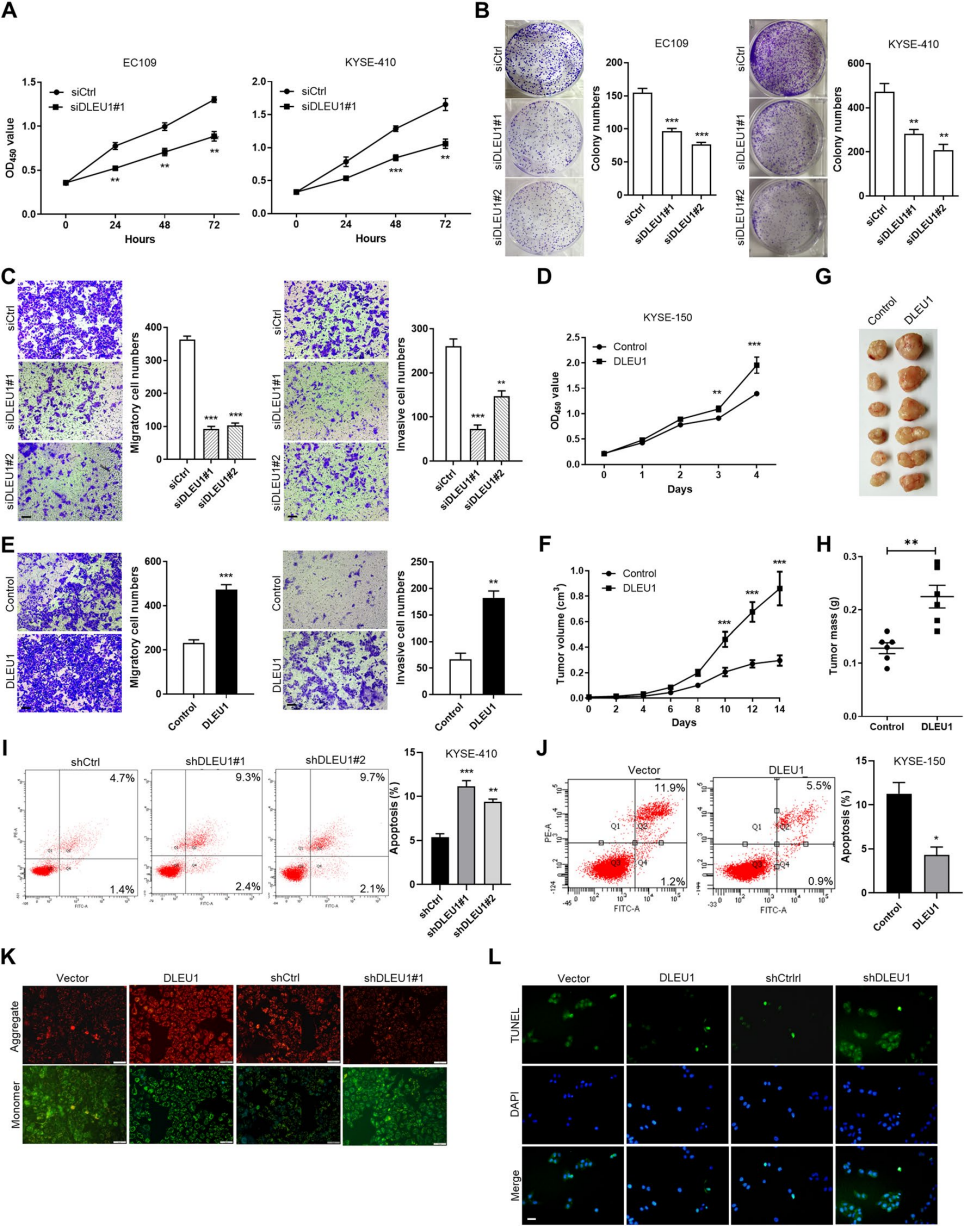
DLEU1 promotes ESCC growth by inhibiting cell apoptosis. **A** The effects of DLEU1 knockdown by siRNA on cell growth were measured by CCK-8 assay. **B** Effects of DLEU1 knockdown on colony formation in EC109 and KYSE-410 cells. **C** Representative images and quantification of DLEU1-silenced KYSE-410 cells that migrated (left) or invaded (right) through the Transwell membrane. Scale bar, 50 μm. **D** The effects of DLEU1 overexpression on cell growth were measured by CCK-8 assay. **E** Representative images and quantification of migratory (left) or invasive (right) DLEU1-overexpressing KYSE-150 cells. Scale bar, 50 μm. **F** Effect of DLEU1 on xenograft tumor growth in nude mice. Tumor volume was measured every other day and calculated. **G** Tumors were excised from mice at the end of the experiment. **H** Tumor weights were measured. **I**, **J** Annexin V/PI flow cytometry analysis of the effects of DLEU1 on cisplatin-induced cell apoptosis in DLEU1-silenced KYSE-410 (**I**) or DLEU1-overexpressing KYSE-150 cells (**J**). **K**, **L** Representative images showing apoptotic ESCC cells measured by JC-1 (**K**) and TUNEL staining (**L**). Scale bar, 50 μm. Significance was determined by two-way ANOVA with Sidak's post-hoc test (**A**, **F**), one-way ANOVA with Tukey's post-hoc test (**B**, **C**, **I**), or unpaired Student's *t* test (**E**, **F**, **H**, **J**). *P < 0.05, **P < 0.01, ***P < 0.001

To clarify the biological significance of DLEU1 in ESCC, EC109 and KYSE-410 cells with high endogenous DLEU1 levels (Fig. 6C) were transfected with specific siRNAs to knockdown DLEU1 expression. We confirmed that knockdown of DLEU1 by two different siRNAs resulted in decreased expression levels of DLEU1 in ESCC cells (Additional file 1: Fig. S1). CCK-8 and colony formation assays demonstrated that silencing DLEU1 significantly decreased the growth capacity and colony formation efficiency (Fig. 2A, B). DLEU1 repression also led to a drastic downregulation of the migratory ability and invasive potential of ESCC cells (Fig. 2C). In contrast, ectopic expression of DLEU1 in KYSE-150 cells with low endogenous DLEU1 levels resulted in a significant enhancement of cell growth (Fig. 2D), migration, and invasion (Fig. 2E). When transplanted into nude mice, the tumor growth of cells with DLEU1 overexpression was significantly promoted compared with that of control cells (Fig. 2F–H). Collectively, these data suggest a growth-promoting role of DLEU1 in ESCC.

The enhancement of growth induced by DLEU1 could reflect either an increased proliferation rate or protection from cell apoptosis. To determine how DLEU1 contributes to the growth of ESCC cells, we first examined the effect of DLEU1 knockdown on cell proliferation using PI staining. No significant changes in cell cycle distribution were observed after DLEU1 knockdown (data not shown), suggesting that silencing DLEU1 had little impact on cell cycle progression. Nevertheless, the annexin V/PI assay showed that knockdown of DLEU1 by shRNAs resulted in a marked increase in cell apoptosis rates in KYSE-410 cells, whereas DLEU1 overexpression inhibited apoptotic cell death in KYSE-150 cells (Fig. 2I, J). The antiapoptotic effect of DLEU1 was further confirmed by mitochondrial membrane potential detection and TUNEL staining (Fig. 2K, L). The results indicated that DLEU1 was required for the survival and tumorigenicity of ESCC cells and played this role by inhibiting apoptosis.

### DLEU1 stabilizes DYNLL1 by preventing its ubiquitination and degradation

**Fig. 3:**
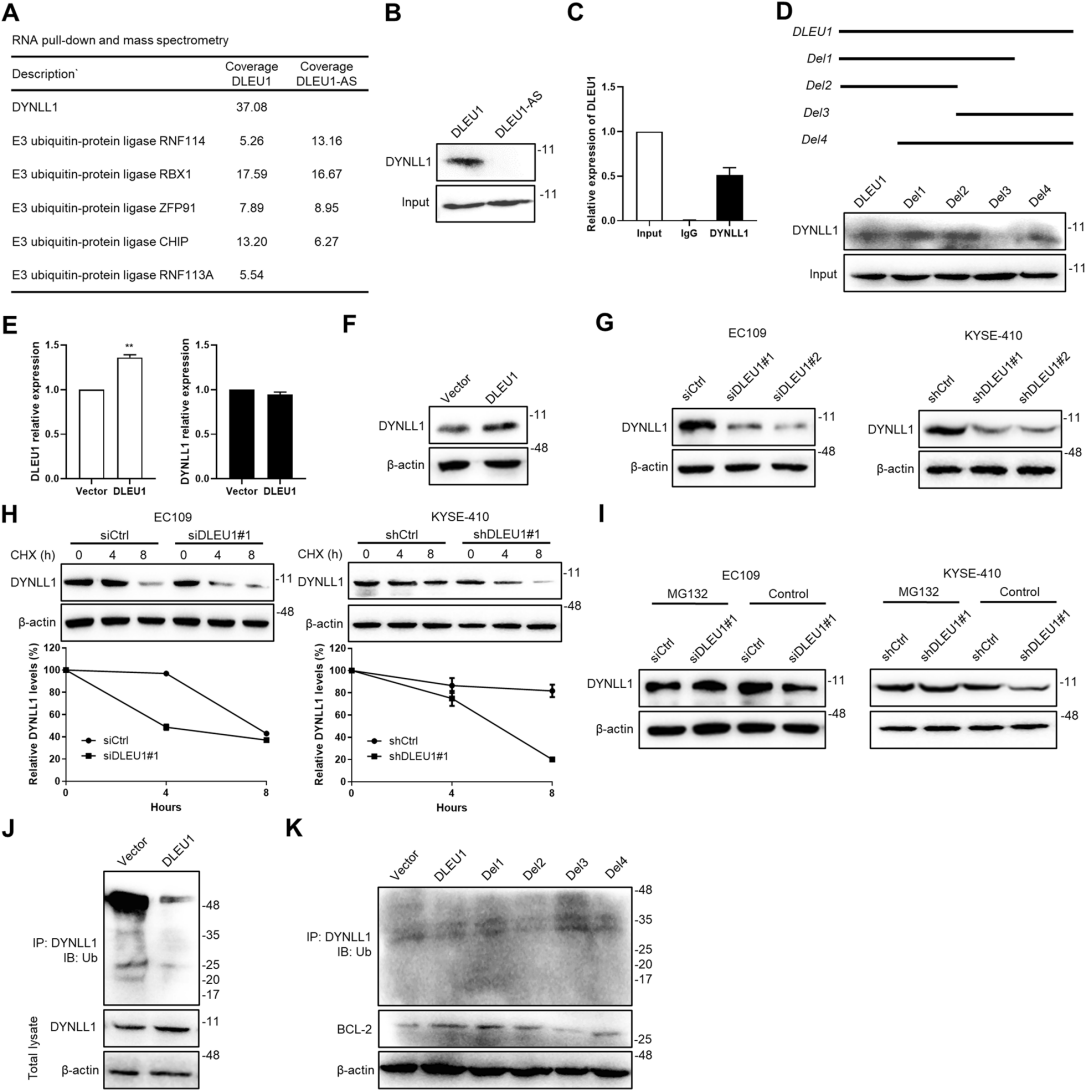
DLEU1 stabilizes the DYNLL1 protein by interfering with its ubiquitination and degradation. **A** Representative proteins identified by RNA pull-down and mass spectrometry using biotinylated DLEU1 sense or antisense (DLEU1-AS) RNA generated in vitro. **B** Immunoblotting analysis of proteins retrieved from the DLEU1 pull-down assay using a DYNLL1 antibody. **C** Total lysates of KYSE-150-DLEU1 cells were subjected to immunoprecipitation with anti-DYNLL1 or anti-IgG antibody followed by qRT-PCR analysis to detect DLEU1 RNA. **D** Immunoblot detection of DYNLL1 protein retrieved from KYSE-150-DLEU1 cells by in vitro transcribed biotinylated RNAs of the indicated DLEU1 constructs. **E** qRT-PCR analysis of DYNLL1 expression in KYSE-150 cells with ectopic DLEU1. **F** Immunoblotting analysis of the DYNLL1 protein in KYSE-150 cells overexpressing DLEU1. β-actin was used as a loading control. **G** DLEU1 depletion decreased the protein levels of DYNLL1. **H** Cells transfected with the indicated siRNAs or shRNAs were treated with CHX (50 mg/mL) for 4 or 8 h before harvest. DYNLL1 protein levels were measured by immunoblotting analysis. β-actin served as a loading control. **I** Cells were cultured in the presence or absence of MG132 (20 μM) after DLEU1 knockdown. DYNLL1 protein levels were checked by immunoblotting. **J** Lysates from KYSE-150 cells with DLEU1 overexpression and MG132 treatment were subjected to immunoprecipitation with anti-DYNLL1 antibody followed by immunoblotting with ubiquitin antibody. **K** KYSE-150 cells were transfected with full-length or truncated mutants of DLEU1 and were subjected to immunoprecipitation followed by immunoblotting analysis with the indicated antibodies

A number of lncRNAs have been reported to exert their biological function by forming complexes with proteins [21]. We therefore performed an RNA pull-down assay followed by mass spectrum analysis to identify DLEU1-associated proteins and elucidate the potential mechanisms of DLEU1 in ESCC. Among the identified DLEU1-binding proteins, we selected DYNLL1 based on the functional annotation of precipitated proteins (Fig. 3A). Dynein light chain LC8-type 1 (DYNLL1, also known as DLC1 and LC8) is a hub protein that has been proposed to regulate mitochondrial apoptosis [22]. The association of DYNLL1 with DLEU1 sense RNA, but not antisense RNA, was further verified by immunoblotting (Fig. 3B). We also performed RNA immunoprecipitation analysis of total cell lysates with the anti-DYNLL1 antibody. qRT-PCR analysis of the immunoprecipitates demonstrated that DYNLL1 was associated with DLEU1 RNA (Fig. 3C). To identify which region was required for the association of DLEU1 with DYNLL1, we constructed a series of DLEU1 deletion mutants named Del1 (1–1086 nt), Del2 (1–724 nt), Del3 (725–1448 nt), and Del4 (363–1448 nt). RNA fragments were transcribed in vitro from these deletion mutants and used for RNA pull-down assays. Immunoblotting analysis of the DYNLL1 protein showed that the DLEU1 construct Del3 nearly completely lost its ability to bind DYNLL1, suggesting that the region was not essential for DLEU1 binding to DYNLL1 (Fig. 3D).

Next, we explored whether DLEU1 could regulate the expression of DYNLL1 in ESCC cells. No significant change in DYNLL1 transcription was observed in DLEU1-overexpressing KYSE-150 cells compared with vector control cells (Fig. 3E). Nevertheless, DYNLL1 protein levels were markedly increased after ectopic DLEU1 expression in KYSE-150 cells but drastically decreased following knockdown of DLEU1 by siRNAs or shRNAs in EC109 and KYSE-410 cells (Fig. 3F, G), suggesting that DLEU1 could enhance DYNLL1 expression by posttranscriptional mechanisms. The protein synthesis inhibitor cycloheximide (CHX) was used to evaluate the effect of DLEU1 on the degradation of DYNLL1. We found that knockdown of DLEU1 shortened the half-life of DYNLL1 in EC109 and KYSE-410 cells (Fig. 3H). Moreover, inhibition of proteasome activity with MG132 prevented the downregulation of DYNLL1 protein caused by DLEU1 silencing (Fig. 3I). We found that ectopic expression of DLEU1 in KYSE-150 cells increased the stability of DYNLL1 by suppressing its ubiquitination (Fig. 3J). In contrast, a higher ubiquitination level of DYNLL1 was observed in cells expressing DLEU1-Del3, which was unable to associate with DYNLL1 (Fig. 3D), compared with the full-length and other deletion fragments of DLEU1 (Fig. 3K). Together, these results demonstrated that DLEU1 increased the stability of DYNLL1 by inhibiting its ubiquitin–proteasome-mediated degradation.

### DLEU1 blocks RNF114-mediated DYNLL1 ubiquitination

**Fig. 4:**
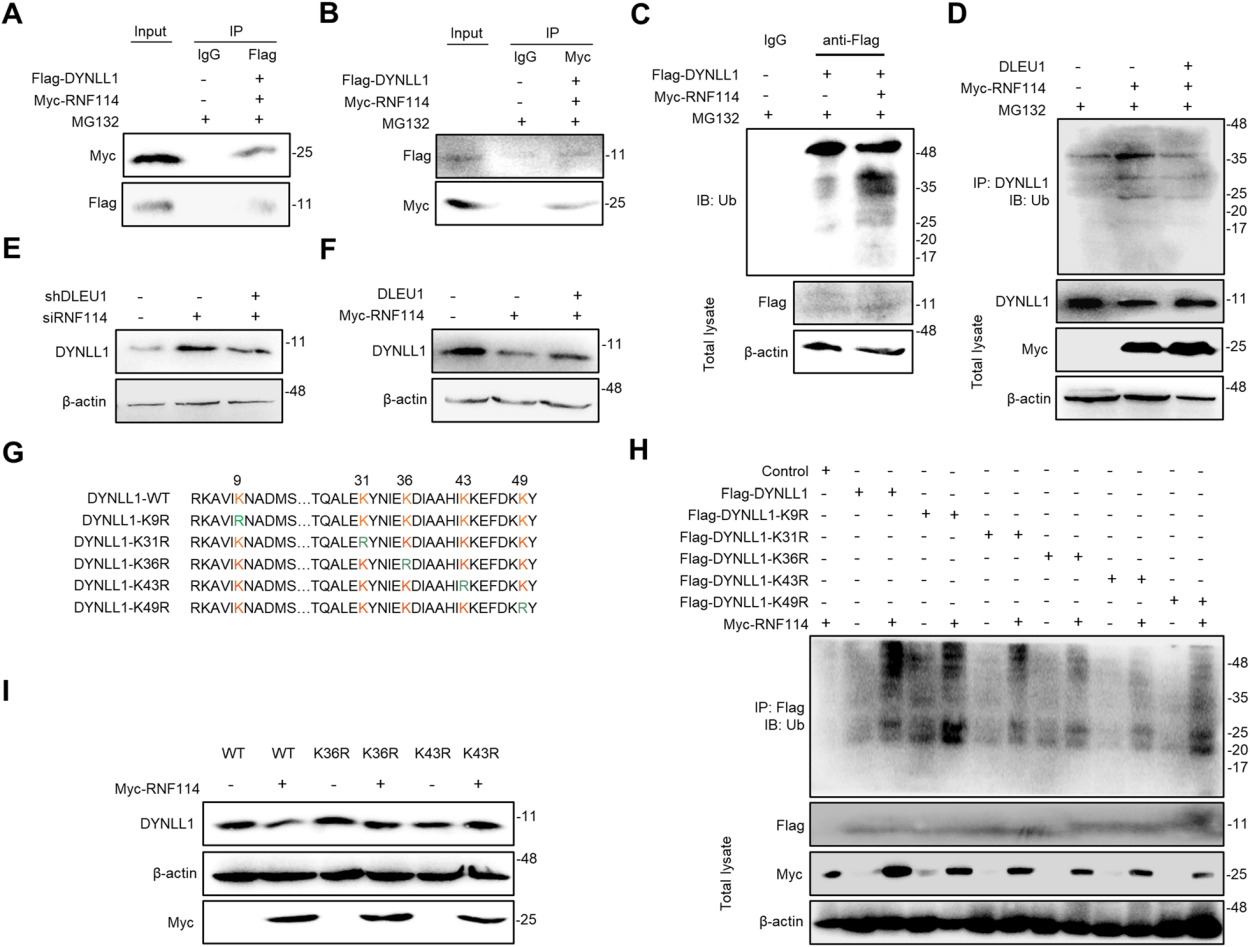
DLEU1 inhibits RNF114-mediated ubiquitination of DYNLL1. **A**, **B** KYSE-150-DLEU1 cells transfected with Flag-tagged DYNLL1 and Myc-tagged DYNLL1 and treated with MG132 were immunoprecipitated with anti-Flag (**A**) or anti-Myc (**B**) antibody followed by immunoblotting with anti-Myc or anti-Flag antibody. **C** Lysates from KYSE-150-DLEU1 cells transfected with the indicated expression constructs and treated with MG132 were subjected to immunoprecipitation with anti-Flag antibody followed by immunoblotting with ubiquitin antibody. **D** Lysates from KYSE-150-DLEU1 cells transfected with the indicated expression constructs and treated with MG132 were subjected to immunoprecipitation with anti-DYNLL1 antibody followed by immunoblotting with ubiquitin antibody. **E** Lysates from KYSE-410 cells transfected with siRNF114 and/or shDLEU1 were subjected to immunoblotting with a DYNLL1 antibody. β-actin was used as a loading control. **F** Lysates from KYSE-150 cells transfected with Myc-RNF114 and/or DLEU1 were subjected to immunoblotting with anti-DYNLL1. **G** Sequences of wild-type and mutated DYNLL1. **H** Lysates from KYSE-150 cells transfected with the indicated expression constructs and treated with MG132 were immunoprecipitated with anti-Flag antibody followed by immunoblotting with ubiquitin antibody. **I** Lysates from KYSE-150 cells transfected with the indicated expression constructs were subjected to immunoblotting analysis. β-actin served as a loading control

Ubiquitin ligase E3, the critical factor of the ubiquitin–proteasome system for selective degradation, can recognize proteins that will be degraded and attach ubiquitin to target proteins [21, 23]. To identify the ubiquitin ligase targeting DYNLL1 in ESCC cells, we reanalyzed the DLEU1 mass spectrometry data and searched for coprecipitated E3 ubiquitin ligases. We selected RNF114 as the candidate since its coverage in the DLEU1 group was markedly lower than that in the DLEU1 antisense group (Fig. 3A). Pull-down assays revealed that RNF114 coprecipitated with DYNLL1 and vice versa (Fig. 4A, B), suggesting that RNF114 is the ubiquitin ligase targeting DYNLL1 for degradation. Indeed, overexpression of RNF114 led to an increased ubiquitination level of DYNLL1 (Fig. 4C). Furthermore, DLEU1 overexpression abolished the ubiquitination of DYNLL1 induced by RNF114 and restored DYNLL1 expression (Fig. 4D). We found that DLEU1 knockdown abrogated siRNF114-induced DYNLL1 expression (Fig. 4E). Conversely, ectopically expressed DLEU1 restored the RNF114-induced degradation of DYNLL1 (Fig. 4F). These results indicated that RNF114 mediated DYNLL1 ubiquitination and degradation, while DLEU1 could interfere with the degradation of DYNLL1 mediated by RNF114.

To identify specific sites where the DYNLL1 protein is ubiquitinated, we generated mutant derivatives of DYNLL1 in which Lys-9, -31, -36, -43, or -49 were replaced by Arg (DYNLL1-K9R, -K31R, -K36R, -K43R, or K49R; Fig. 4G) and examined their ubiquitination in the presence of ectopic RNF114 in ESCC cells. All DYNLL1 derivatives underwent RNF114-mediated ubiquitination except for DYNLL1-K36R and DYNLL1-K43R, whose ubiquitination was attenuated (Fig. 4H). In addition, we found that overexpression of RNF114 did not induce the degradation of DYNLL1-K36R or DYNLL1-K43R (Fig. 4I). These results indicated that DYNLL1 might be ubiquitinated at Lys-36 and Lys-43 by RNF114.

### The DLEU1/DYNLL1 axis modulates apoptotic signaling

**Fig. 5:**
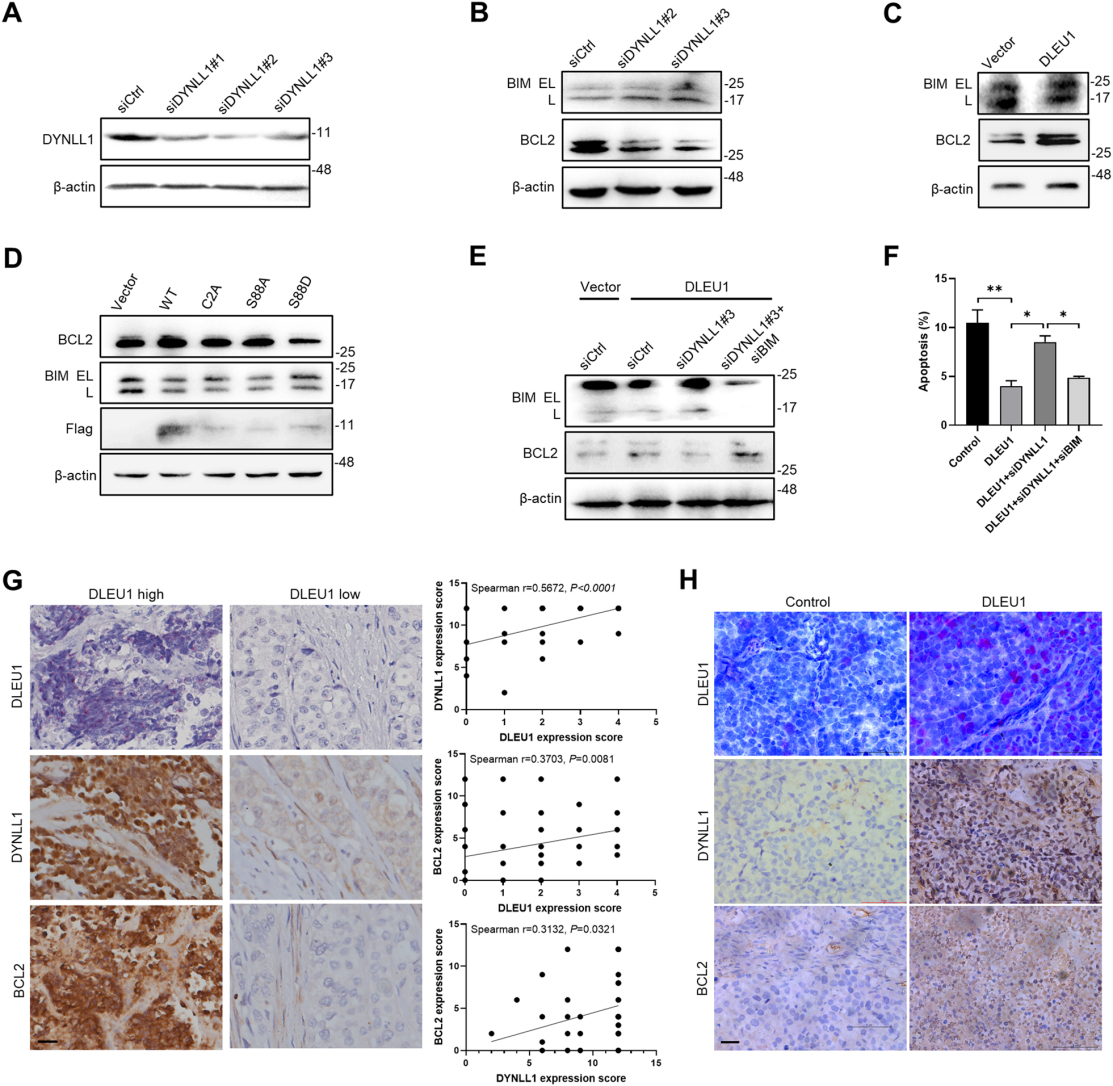
The DLEU1/DYNLL1 axis modulates mitochondrial apoptotic signaling. **A** DYNLL1 was successfully knocked down in KYSE-150-DLEU1 cells. β-actin was used as a loading control. **B** Immunoblotting analysis of BIM and BCL2 expression after DYNLL1 depletion in KYSE-150-DLEU1 cells. **C** Immunoblotting analysis of BIM and BCL2 expression after DLEU1 overexpression in KYSE-150 cells. **D** Cells transfected with wild-type or DYNLL1 mutants were subjected to immunoblotting analysis with the indicated antibodies. **E** The effects of concomitant silencing of DYNLL1 and BIM on BCL2 expression were evaluated by immunoblotting in KYSE-150 cells with ectopic DLEU1 expression. **F** Annexin V/PI flow cytometry analysis of the effects of DYNLL1 and BIM depletion on cisplatin-induced cell apoptosis in DLEU1-overexpressing KYSE-150 cells. Significance was assessed by one-way ANOVA with Tukey’s post-hoc test. *P < 0.05, **P < 0.01. **G** Representative images (left) and scatter plot (right) showing a positive correlation among DLEU1, DYNLL1, and BCL2 in clinical ESCC specimens (n = 38). Scale bar, 50 μm. **H** Representative immunohistochemical staining of DYNLL1 and BCL2 proteins in DLEU1-overexpressing ESCC xenografts. Scale bar, 50 μm

DYNLL1 has been proposed to limit the proapoptotic activity of BIM and contribute to the efficient expression of the antiapoptotic protein BCL2 [22]. We therefore asked whether this cascade is involved in the modulation of apoptotic cell death by DLEU1 in ESCC. Immunoblotting demonstrated that the level of BCL2 was strongly downregulated, while BIM expression was somewhat increased in KYSE-150-DLEU1 cells upon DYNLL1 knockdown (Fig. 5A, B). In contrast, ectopic DLEU1 expression upregulated the expression of the antiapoptotic protein BCL2 (Fig. 5C). The residues Cys2 and Ser88 of DYNLL1 have been implicated in affecting its interaction network with other factors [24, 25]. To illustrate the functional sites of DYNLL1, mutation of Cys2 to alanine (C2A), phospho-null mutation of Ser88 (S88A), and phosphomimetic Ser88 mutation (S88D) constructs were generated. Immunoblotting analysis revealed that DYNLL1-S88D resulted in slightly higher BIM expression but decreased BCL2 expression compared with wild-type DYNLL1 and other mutants in KYSE-150 cells (Fig. 5D). siDYNLL1-induced changes in BCL2 and BIM expression levels could be reversed by BIM knockdown (Fig. 5E). Consistently, DYNLL1 knockdown mitigated the promoting effect of DLEU1 on cell survival, while the concomitant loss of BIM completely abolished the effect of DYNLL1 silencing on apoptotic cell death (Fig. 5F).

To establish whether the expression levels of DLEU1, DYNLL1, and BCL2 are clinically associated, we evaluated DLEU1 RNA expression by RNAscope and evaluated the protein levels of DYNLL1 and BCL2 by immunohistochemistry staining in ESCC tissues. DYNLL1 and BCL2 protein levels were positively correlated with DLEU1 levels in ESCC samples (r = 0.567 and 0.370). A positive correlation between DYNLL1 and BCL2 levels was also observed (r = 0.313; Fig. 5G). Consistently, histological examination of xenograft tumors in nude mice demonstrated higher DYNLL1 and BCL2 protein levels in the DLEU1-overexpressing group than in the control group (Fig. 5H). Collectively, these data demonstrated that DLEU1/DYNLL1 axis-mediated upregulation of BCL2 was involved in the survival of ESCC cells.

### DLEU1 upregulation is inversely associated with promoter methylation

**Fig. 6:**
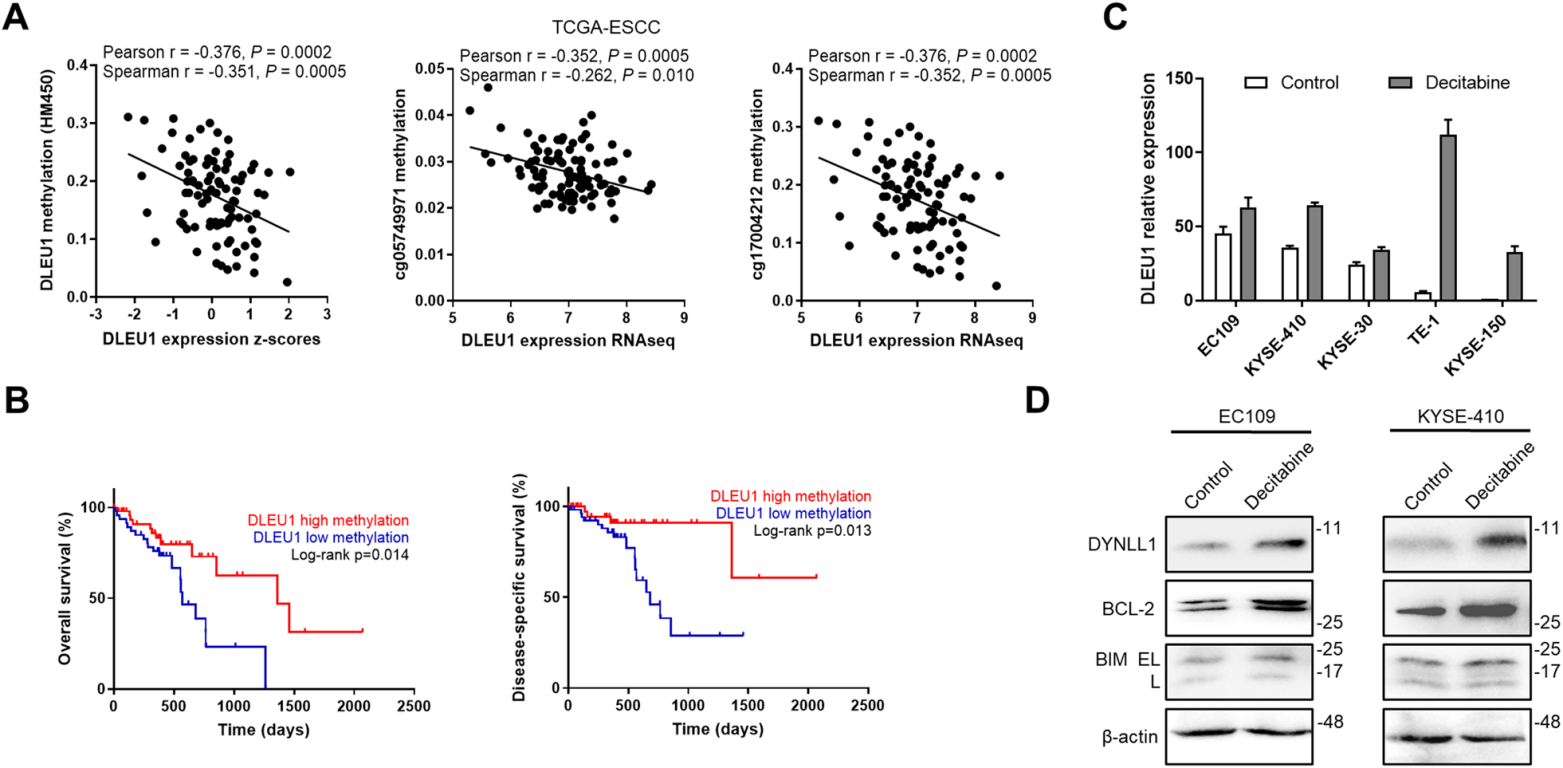
DLEU1 expression is epigenetically regulated in ESCC. **A** Scatter plots presenting the negative correlation between DLEU1 expression levels and its promoter methylation values in the TCGA-ESCC dataset. **B** Kaplan–Meier curves of overall survival (left) and disease-specific survival according to DLEU1 DNA methylation in ESCC patients from the TCGA project. **C** Relative DLEU1 expression levels in a panel of ESCC cell lines cultured in the presence or absence of decitabine (0.5 μM) for 48 h. **D** Immunoblotting analysis of the indicated proteins in EC109 and KYSE-410 cells treated with decitabine. β-actin was used as a loading control

To decipher the underlying mechanisms of dysregulated DLEU1 expression in ESCC, we explored tumor-intrinsic oncogenic events in the DLEU1 gene locus. Genetic alterations of the DLEU1 locus in the TCGA-ESCC cohort were first analyzed (Additional file 1: Fig. S2A). The DLEU1 gene showed rare alterations (2.1%, 2/96 cases with deep deletions). A positive correlation of DLEU1 RNA expression with its gene copy number was observed in ESCC samples, suggesting that the upregulation of DLEU1 was partly due to DNA copy number gain (Additional file 1: Fig. S2B, C). DNA methylation was found to be one of the causes of abnormal gene expression, which promoted the genesis of tumors. Intriguingly, DLEU1 expression was negatively correlated with the DNA methylation level of global or individual CpG sites within the DLEU1 promoter region in ESCC patients (Fig. 6A). In addition, Kaplan–Meier analysis revealed that ESCC patients with low DLEU1 methylation had significantly shortened overall survival and disease-specific survival compared with those with high DLEU1 methylation (Fig. 6B). Treatment with the DNA methyltransferase inhibitor decitabine significantly enhanced DLEU1 expression in a series of ESCC cell lines (Fig. 6C). Consistently, DNA demethylation by decitabine enhanced DYNLL1 and BCL2 protein levels in EC109 and KYSE-410 cells (Fig. 6D). These findings collectively indicated that the upregulation of DLEU1 in ESCC was facilitated by DNA copy number gain and promoter hypomethylation.

### Targeting DLEU1 sensitizes ESCC cells to cisplatin-induced death

**Fig. 7:**
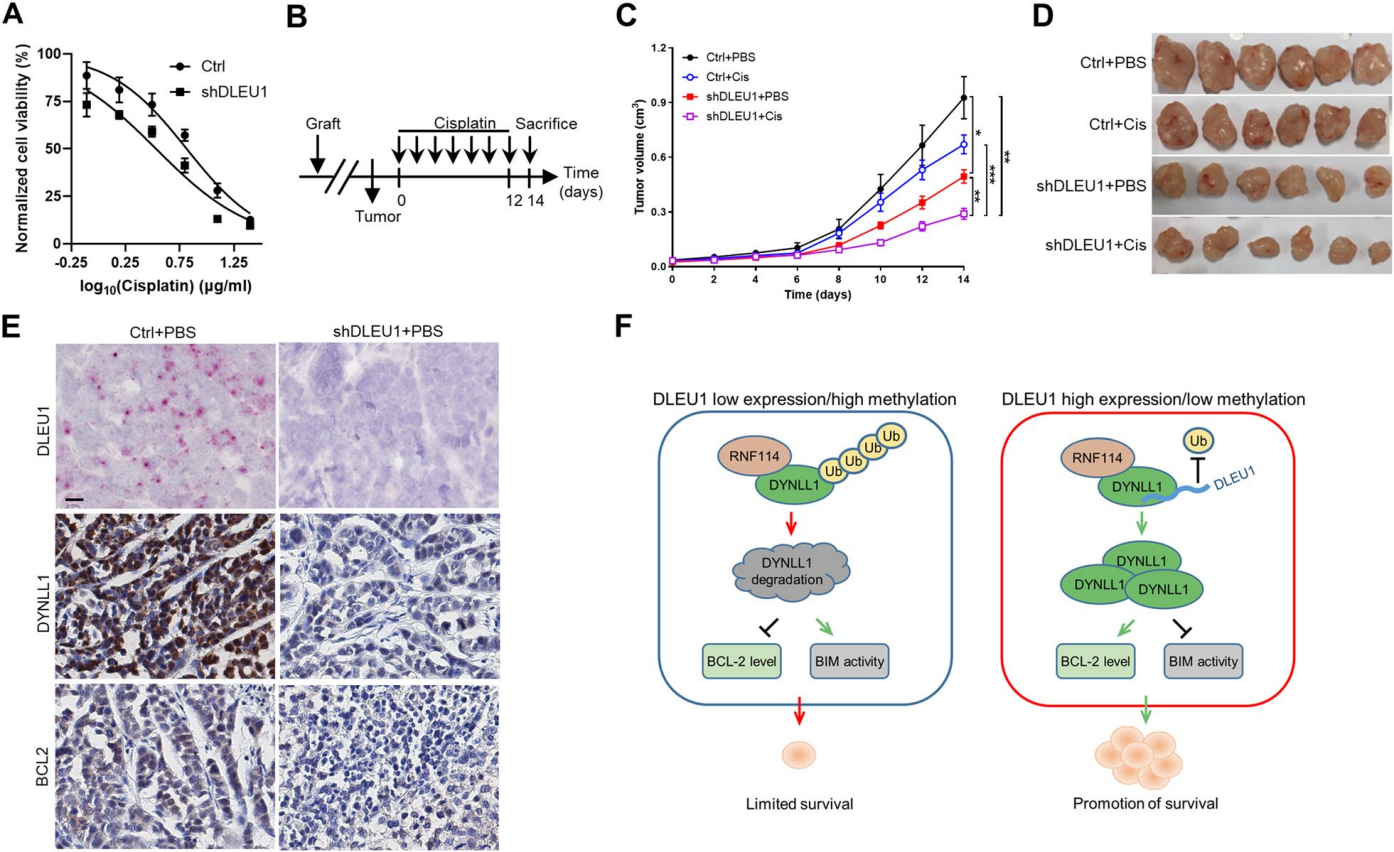
DLEU1 depletion sensitizes ESCC cells to cisplatin-induced death. **A** Dose–response curves of KYSE-410 cells to cisplatin after DLEU1 knockdown. **B** Schematic diagram showing the experimental strategy (n = 6 per group). **C** Tumor volume of the ESCC xenografts was measured. Significance was determined by two-way ANOVA with Tukey’s post-hoc test. *P < 0.05, **P < 0.01, ***P < 0.001. **D** Tumors were excised from nude mice at the end of the experiment. **E** Representative immunohistochemical staining of DYNLL1 and BCL2 proteins in DLEU1-silencing ESCC xenografts. Scale bar, 50 μm. **F** Graphical abstract illustrating the mechanism by which DLEU1 stabilizes DYNLL1 and promotes cell survival in ESCC

In line with our above finding that DLEU1 could prevent apoptotic cell death, knockdown of DLEU1 enhanced the antitumor effect of cisplatin treatment in KYSE-410 cells (Fig. 7A). To further explore the biological significance of DLEU1 in supporting tumor development, mice with established xenografts with or without DLEU1 silencing were randomized to receive treatment with vehicle control or cisplatin (Fig. 7B). DLEU1 knockdown or cisplatin treatment alone was sufficient to impair tumor growth. Moreover, compared with cisplatin alone, the combination of cisplatin and DLEU1 knockdown exhibited a stronger inhibitory effect on xenograft tumor growth in nude mice (Fig. 7C, D). Histological examination of xenograft tumors showed that DLEU1 silencing was accompanied by decreased DYNLL1 and BCL2 protein levels (Fig. 7E). These results suggested that targeting DLEU1 could increase the sensitivity of ESCC cells to cisplatin therapy.

## Discussion

Clinically, surgical resection is the major treatment for esophageal cancer, while radiotherapy and chemotherapy are the primary adjuvant treatments. However, outcomes are still unsatisfactory due to the limited efficacy and severe adverse effects of conventional treatments [7]. Thus, elucidating the carcinogenesis mechanisms and identifying new biomarkers and therapeutic targets could be quite beneficial to optimizing the current therapeutic regimens [26]. Increasing evidence has shown that lncRNAs play important roles in the development and progression of multiple human cancers, including esophageal cancer [27, 28]. Dysregulated lncRNAs, such as UCA1, HOTAIR, and TUG1, have been proposed as prognostic biomarkers for esophageal cancer [29–31]. In this study, our findings showed that lncRNA DLEU1 was significantly upregulated in ESCC tissues and correlated with clinical severity and poor prognosis, suggesting the clinical value of DLEU1 in ESCC. High expression of DLEU1 promoted ESCC growth in vitro and in vivo. Moreover, we observed that DLEU1 could inhibit the apoptosis of ESCC cells but had little effect on the cell cycle, indicating that DLEU1 plays a significant role in promoting tumor growth mainly by inhibiting cell apoptosis. Consistently, DLEU1 has been demonstrated to be dysregulated and exerts an oncogenic function in tumors such as oral squamous cell carcinoma [19], glioma [32], endometrial cancer [33], and non-small-cell lung cancer [34]. Although bioinformatic analyses have indicated the association of DLEU1 with unfavorable clinical outcomes, further work in clinical specimens with prognostic information is required to better understand the biological functions of DLEU1 in ESCC.

DLEU1 has multiple mechanisms of action in tumors. DLEU1 promotes tumor progression and chemotherapy resistance in bladder cancer by regulating the miR-99b/HS3ST3B1 axis [35]. DLEU1 exacerbates pancreatic ductal adenocarcinoma through the miR-381/CXCR4 axis [36]. DLEU1 can also promote papillary thyroid carcinoma progression by sponging miR-421 and thus increasing ROCK1 expression [37]. On the other hand, DLEU1 can function as a molecular mediator in transcriptional and posttranscriptional processes. DLEU1 contributes to cell proliferation by recruiting LSD1 to epigenetically suppress *KLF2* in gastric cancer [38]. DLEU1 recruits SMARCA1 to epigenetically activate the *KPNA3* gene in colorectal cancer, thereby promoting cell proliferation and migration [18]. Here, we performed experimental screening through RNA pull-down and mass spectrometry and identified DYNLL1, a widely expressed and highly conserved multifunctional protein [25, 39, 40], as a critical interacting target of DLEU1. Although the 725–1448 nt region (Del3) of DLEU1 was determined to not be required for binding with the DYNLL1 protein, the precise region mediating the binding between DLEU1 and DYNLL1 needs further investigation.

During B cell development, DYNLL1 expression could be activated by ASCIZ to restrain the proapoptotic activity of Bim in immature B cells, highlighting the importance of DYNLL1 in setting homeostatic survival levels [41]. The B-2 lymphoid cell defect in *Dynll1*-deficient mice was associated with significantly reduced expression of the Bim antagonist BCL2 and could be rescued by codeletion of Bim, suggesting that DYNLL1 is required for efficient Bcl2 expression and to restrain Bim-mediated apoptosis in developing B cells [24]. Moreover, the loss of DYNLL1 dramatically delays the development and expansion of MYC-driven B-cell lymphoma in mice due to increased BIM-mediated apoptotic cell death, although BIM levels are not elevated [42]. Nevertheless, RNAi against DYNLL1 leads to reduced Bim levels to varying degrees in HeLa epithelial cells and 1205Lu melanoma cells [43]. In breast cancer, overexpression of DYNLL1 protects cells from UV-induced apoptosis and promotes cancerous properties [44]. A Ser88 to alanine (S88A) mutation of DYNLL1 does not affect Bim_L_ binding, while a Ser88 to glutamic acid (S88E) mutation, which mimics the phosphorylated form of DYNLL1, impairs its interaction with Bim_L_ in mammary epithelial cells [39, 44]. In contrast, the phosphomimetic Ser88 to aspartic acid (S88D) mutation showed a clear enhanced interaction with DNA end-resection enzymes such as MRE11 in ovarian cancer [45]. Consistent with previous reports, our results suggested that DLEU1 functions through DYNLL1 to restrain the proapoptotic activity of BIM and upregulate the antiapoptotic BCL2 protein to inhibit apoptotic cell death in ESCC.

In our study, promoter hypomethylation led to the upregulation of DLEU1 expression in ESCC. In line with our findings, a recent report suggests that decreased DNA methylation and increased histone modifications may contribute to DLEU1 upregulation in cancer [46]. Drug resistance is the main reason for the failure of chemotherapy. Recent evidence indicates that DLEU1 could promote cisplatin resistance in bladder cancer and nasopharyngeal carcinoma [35, 47]. Consistently, our study provided evidence that targeting DLEU1 increased the sensitivity of ESCC cells to cisplatin treatment.

## Conclusions

In summary, our findings show that the lncRNA DLEU1 exerts a survival-promoting function in ESCC by interfering with the RNF114-mediated ubiquitination and proteasomal degradation of DYNLL1 (Fig. 7F). DLEU1/DYNLL1 signaling may be a promising therapeutic target for ESCC.

## Supplementary Information

**Additional file 1**: **Figure S1. **Validation of the DLEU1 knockdown efficacy by siRNAs or shRNAs in EC109 cells. **Figure S2. **Association of DLEU1 expression level with its copy number status. (A) OncoPrint showing DLEU1 copy number alteration and mutation across the ESCC samples in the TCGA-ESCC dataset. (B) DLEU1 expression levels in ESCC patients with the indicated putative copy number alterations derived from the cBioPortal for Cancer Genomics. (C) The scatter plot revealed a positive correlation between DLEU1 expression and its relative copy number. **Table S1. **Oligonucleotide sequences. **Table S2. **Correlation between DLEU1 expression and clinicopathological features in the TCGA-ESCC dataset.

## Data Availability

The data sets analysed during this study are available in public, open access repositories listed in this article.
