# Supplementary material for: DLEU1 promotes cell survival by preventing DYNLL1 degradation in esophageal squamous cell carcinoma
Source: J Transl Med. 2022 May 26;20:245. doi: 10.1186/s12967-022-03449-w (PMC9134706; doi:10.1186/s12967-022-03449-w)
Supplement: Supplementary file 1 — Additional file 1: Figure S1. Validation of the DLEU1 knockdown efficacy by siRNAs or shRNAs in EC109 cells. Figure S2. Association of DLEU1 expression level with its copy number status. (A) OncoPrint showing DLEU1 copy number alteration and mutation across the ESCC samples in the TCGA-ESCC dataset. (B) DLEU1 expression levels in ESCC patients with the indicated putative copy number alterations derived from the cBioPortal for Cancer Genomics. (C) The scatter plot revealed a positive correlation between DLEU1 expression and its relative copy number. Table S1. Oligonucleotide sequences. Table S2. Correlation between DLEU1 expression and clinicopathological features in the TCGA-ESCC dataset. [file 12967_2022_3449_MOESM1_ESM.docx]

**
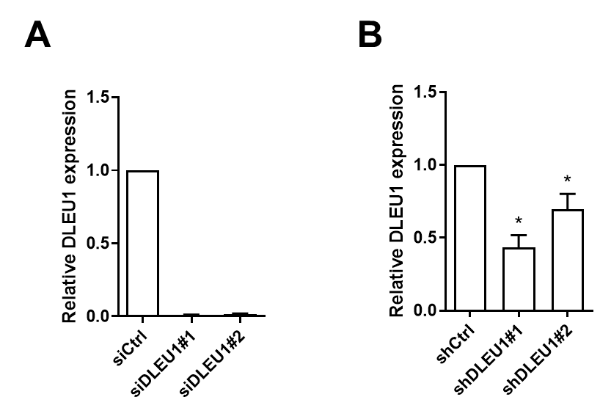
**

**Figure S1.** Validation of the DLEU1 knockdown efficacy by siRNAs or shRNAs in EC109 cells.

**
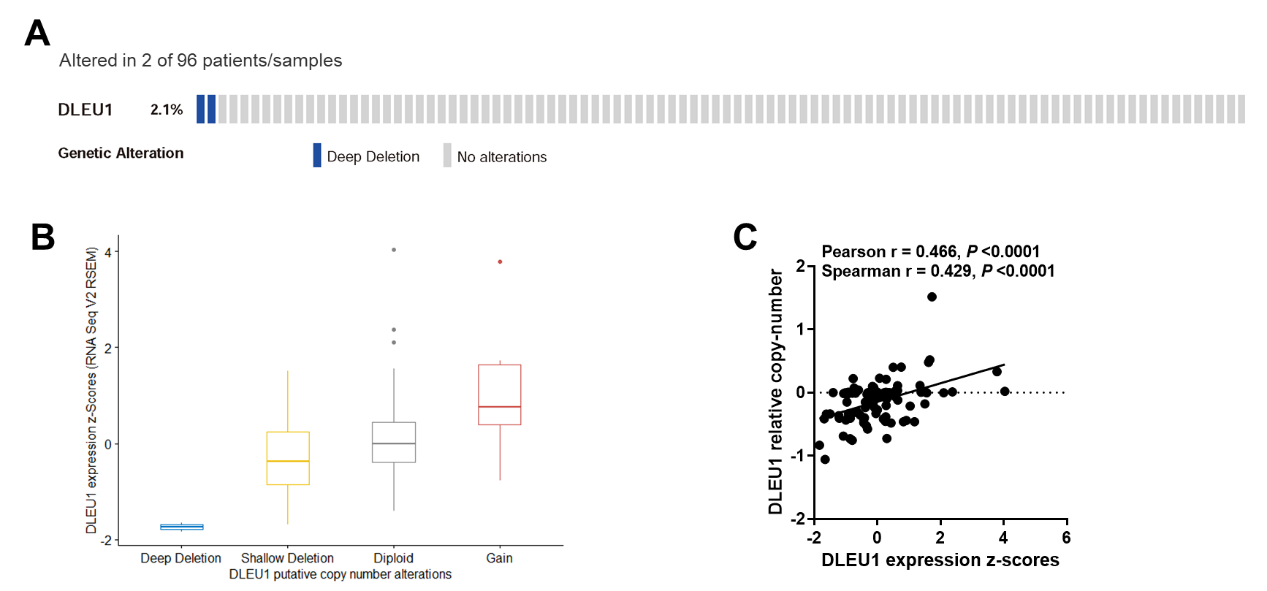
 Figure S2.** Association of DLEU1 expression level with its copy number status. (A) OncoPrint showing DLEU1 copy number alteration and mutation across the ESCC samples in the TCGA-ESCC dataset. (B) DLEU1 expression levels in ESCC patients with the indicated putative copy number alterations derived from the cBioPortal for Cancer Genomics. (C) The scatter plot revealed a positive correlation between DLEU1 expression and its relative copy number.

**Table S1.** Oligonucleotide sequences.

| **Primers for real-time PCR** | | |
| --- | --- | --- |
| DLEU1 | CACGTGCATTTAAAACCGCC | AAGACTTTGGGGCAGATTTCTT |
| GAPDH | GCACCGTCAAGGCTGAGAAC | TGGTGAAGACGCCAGTGGA |
| **Primers for RNA pull-down** | | |
| DLEU1 | TAATACGACTCACTATAGGGAGGCACATGCGCAGAATCAT | AATATTTATAGCTTATGTTAATTCTTATCCCG |
| DLEU1-AS | TAATACGACTCACTATAGGGAATATTTATAGCTTATGTTAATTCTTATCCCG | AGGCACATGCGCAGAATCAT |
| **Sequences for gene knockdown** | | |
| siLINC01614 | GCCAUUUCCCAAAUGUCAATT | UUGACAUUUGGGAAAUGGCTT |
| siLOC284930 | GCUGGGCUUGAUUAUCCUUTT | AAGGAUAAUCAAGCCCAGCTT |
| siKCNMB2-AS1 | CCCAAACAGUGUGGGUCAGTT | CUGACCCACACUGUUUGGGTT |
| siDLEU1#1 | GCAGUCUGUUCUGAACAUATT | UAUGUUCAGAACAGACUGCTT |
| siDLEU1#2 | GGAAACAAACAGACCUAACTT | GUUAGGUCUGUUUGUUUCCTT |
| siDYNLLY1#2 | GAGGAACUUCGGUAGUUAUTT | AUAACUACCGAAGUUCCUCTT |
| siDYNLLY1#3 | GGCCAUUCUUCUGUUCAAATT | UUUGAACAGAAGAAUGGCCTT |
| siBIM | GCCACAAGGUAAUCCUGAATT | UUCAGGAUUACCUUGUGGCTT |

**Table S2.** Correlation between DLEU1 expression and clinicopathological features in the TCGA-ESCC dataset.

| Characteristics | Total | DLEU1 expression | | *P* (χ2) |
| --- | --- | --- | --- | --- |
|  |  | Low | High |  |
| Clinical T |  |  |  |  |
| T1/T2 | 17 | 12 | 5 | 0.175 |
| T3/T4 | 28 | 14 | 14 |  |
| Clinical N |  |  |  |  |
| N0/NX | 29 | 20 | 9 | **0.041*** |
| N1/N2 | 16 | 6 | 10 |  |
| Clinical M |  |  |  |  |
| M0 | 41 | 24 | 17 | 0.741 |
| M1 | 4 | 2 | 2 |  |
| Clinical stage |  |  |  |  |
| I/II | 27 | 19 | 8 | **0.022*** |
| III/IV | 17 | 6 | 11 |  |

Cut-off value: 6.99 log2(norm​_​count+1).
